# Targeting of Mevalonate-Isoprenoid Pathway in Acute Myeloid Leukemia Cells by Bisphosphonate Drugs †

**DOI:** 10.3390/biomedicines10051146

**Published:** 2022-05-16

**Authors:** Emanuela Chiarella, Clelia Nisticò, Anna Di Vito, Helen Linda Morrone, Maria Mesuraca

**Affiliations:** 1 Department of Experimental and Clinical Medicine, University Magna Græcia, 88100 Catanzaro, Italy; nclelia@hotmail.it (C.N.); divito@unicz.it (A.D.V.); 2 Unit of Infectious and Tropical Diseases, Department of Medical and Surgical Sciences, University Magna Græcia, 88100 Catanzaro, Italy; helenlinda.morrone@studenti.unicz.it

**Keywords:** mevalonate pathway, AML, bisphosphonates, small GTPases, protein isoprenylation

## Abstract

Metabolic reprogramming represents a hallmark of tumorigenesis to sustain survival in harsh conditions, rapid growth and metastasis in order to resist to cancer therapies. These metabolic alterations involve glucose metabolism, known as the Warburg effect, increased glutaminolysis and enhanced amino acid and lipid metabolism, especially the cholesterol biosynthesis pathway known as the mevalonate pathway and these are upregulated in several cancer types, including acute myeloid leukemia (AML). In particular, it was demonstrated that the mevalonate pathway has a pivotal role in cellular transformation. Therefore, targeting this biochemical process with drugs such as statins represents a promising therapeutic strategy to be combined with other anticancer treatments. In the last decade, several studies have revealed that amino-bisphosphonates (BP), primarily used for bone fragility disorders, also exhibit potential anti-cancer activity in leukemic cells, as well as in patients with symptomatic multiple myeloma. Indeed, these compounds inhibit the farnesyl pyrophosphate synthase, a key enzyme in the mevalonate pathway, reducing isoprenoid formation of farnesyl pyrophosphate and geranylgeranyl pyrophosphate. This, in turn, inhibits the prenylation of small Guanosine Triphosphate-binding proteins, such as Ras, Rho, Rac, Rab, which are essential for regulating cell survival membrane ruffling and trafficking, interfering with cancer key signaling events involved in clonal expansion and maturation block of progenitor cells in myeloid hematological malignancies. Thus, in this review, we discuss the recent advancements about bisphosphonates’ effects, especially zoledronate, analyzing the biochemical mechanisms and anti-tumor effects on AML model systems. Future studies will be oriented to investigate the clinical relevance and significance of BP treatment in AML, representing an attractive therapeutic strategy that could be integrated into chemotherapy.

## 1. Introduction

In the last few decades, it has become more evident that tumorigenesis is associated with metabolic alterations. Via metabolic reprogramming, cancer cells acquire nutrients to maintain viability and grow in a hypoxic and nutrient deprived environment [1].

At the beginning of the last century, Warburg observed that cancer cells used glucose to generate lactate in vitro, even when there was sufficient oxygen for proper mitochondrial respiration. This phenomenon, termed aerobic glycolysis or the Warburg effect, is considered today to be a key metabolic feature in carcinogenesis [2].

The injury to respiration in cancer cells can be caused by altered growth factors signaling, the activation of HIF-1α-gene transcription in hypoxic or normoxic conditions, genetic alterations activating proto-oncogenes or inducing the loss-of-function of suppressor genes. These genetic impairments increase the expression of glucose transporters and key glycolytic enzymes, accelerating the glycolytic flux [3]. Cancer cells prefer to produce energy through a high conversion rate of glucose to lactate in order to produce ATP faster. However, the lactate produced via fermentation reduces extracellular pH, contributing to microenvironment acidosis, which in turn synergistically enhances tumor invasion and metastasis, and confers resistance to antitumor treatments [4].

**Figure 1:**
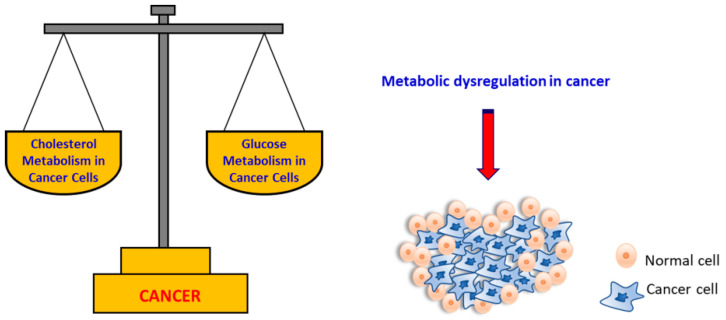
Metabolic reprogramming in cancer involves glucose and cholesterol metabolism alterations. The energy production from fats has the same *weight* as alterations in glucose metabolism in supporting the viability of cancer cells.

Although the Warburg effect plays a central role in cancer progression, cholesterol metabolism has also emerged as a hallmark of cancer development and tumor formation [5] (Figure 1).

Functionally, cholesterol homeostasis is required for normal growth and development of eukaryotic cells [6]. Cholesterol is critical for the synthesis of biological membranes and the modulation of their fluidity and is a plasma lipoprotein constituent, serving as a form of energy storage in animals [7,8]. It is involved in many other functions, such as synthesizing bile acid, producing hormones and is a precursor of Vitamin D [9]. In addition, it is involved in sperm development and immune system defense, as well as in the neural circuit development and functioning in the brain [10,11,12].

Altered cholesterol metabolism has received increasing attention due its role in carcinogenesis [13]. The crystallization of cholesterine from living cells, as well as the alteration in blood cholesterol levels, is a critical phenomenon associated to malignancy, as evidenced from the early 1900s [14,15].

Biochemical and molecular studies have recently reported high cholesterol content in many malignancies including breast, thyroid, uterine, ovarian and renal tumor tissues [16,17,18].

Cancer cells rapidly proliferate in a cholesterol-rich environment and continue to take up and metabolize cholesterol and/or to up-regulate cholesterol biosynthesis for their energy requirements [13]. Feedback loop mechanisms for controlling plasma and intracellular cholesterol homeostasis, as well as cholesterol uptake through the low-density lipoprotein receptor (LDLR) pathway, are dysregulated in many cancer cells compared to normal cells/tissues [19].

Elevated LDLR protein expression accelerates LDL cholesterol-uptake in some highly proliferative cancer cells, suggesting that this more energy-saving process is preferable to cholesterol biosynthesis [9]. LDLR overexpression enhances LDL cholesterol uptake by cells that are actively undergoing growth and proliferation, processes which, in turn, also increase the demand for cholesterol and other lipids for energy [20,21].

Conversely, the downregulation/inhibition of LDLR efficiently blocks cholesterol uptake, improving the chemotherapy efficacy [22].

In many cancers, the mechanisms promoting deregulation of cholesterol homeostasis can be severely altered and induce tumor initiation and progression [23].

For example, the phosphatidylinositol 3-kinase (PI3K)/AKT/mammalian target of rapamycin (mTOR) axis alters cancer enhancing tumor cell demand for cholesterol [24]. Additionally, cholesterol biosynthesis regulation is affected byTP53 gene mutations [25,26].

Additionally, the downregulation/inhibition of lipid droplets (LDS) efficiently blocks cholesterol biosynthesis, improving the radiotherapy efficacy [27].

The significance of dysregulation in cholesterol metabolic pathways associated with malignant progression has become attractive to target cancer cells. Drug combinations are, therefore, used to target cholesterol biosynthesis and cholesterol uptake by sensitizing cancer cells to therapy. Several drugs target the cholesterol synthesis or mevalonate pathway at different levels [28].

For example, statins display anticancer properties with varying sensitivity based on the type of tumor and are drugs widely used in lowering serum cholesterol by inhibiting HMGCR enzyme activity, which catalyzes the rate-limiting step in cholesterol synthesis [29].

Statins have been shown to inhibit proliferation and survival of various cancer cells, as well as reduce metastasis in vivo alone or in combination with other drugs [30].

Moreover, similar to statins, amino-bisphosphonates have shown potential anti-cancer activity in different cancer cell lines including ovarian, colon and hepatic cells [9].

Bisphosphonates block enzymes along the mevalonate pathway, causing prenylation inhibition of small GTP-binding proteins, such as Ras, Rho, Rac, Rab, which are involved in regulating cell survival, membrane ruffling and trafficking [31].

In the last few decades, bisphosphonates have been revealed to be active in leukemic cell lines, as well as in patients with symptomatic multiple myeloma (MM) and against a variety of human tumors [32,33,34,35]. In this review, we discuss the recent advancements in the research about the antileukemic potential of bisphosphonates, especially zoledronic acid (ZOL), in acute myeloid leukemia (AML).

## 2. Biochemistry of the Mevalonate Cascade and Its Regulation in Cholesterol Metabolism

**Figure 2:**
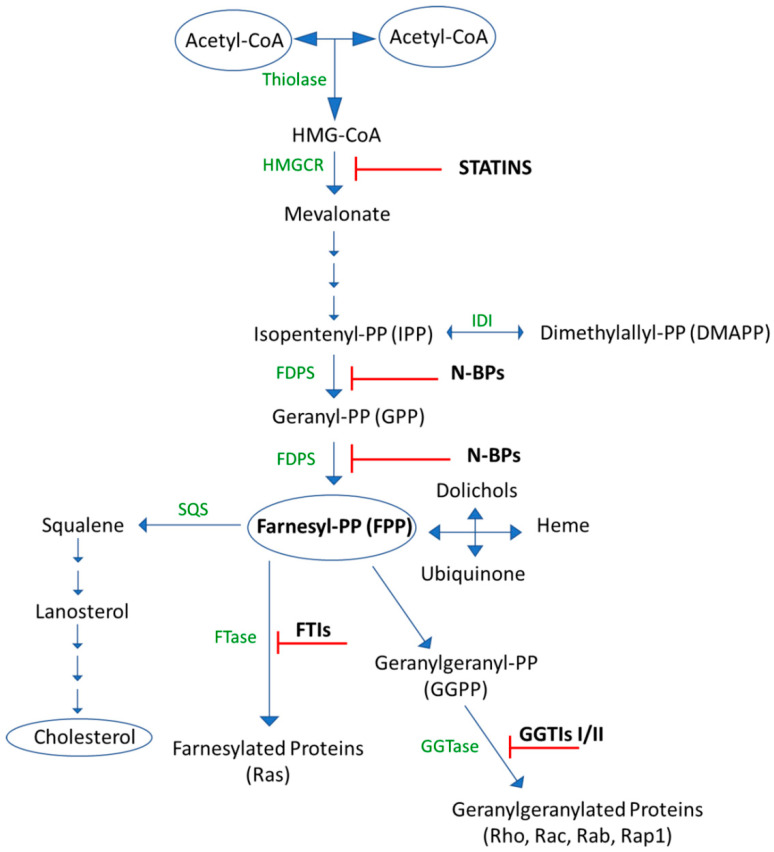
Schematic overview of the mevalonate pathway, isoprenoid/sterol biosynthesis and prenylation of proteins. Statins block HMGCR activity. Nitrogen-containing bisphosphonates (N-BPs) inhibit FDPS activity. Farnesyl transferase inhibitors (FTIs) and geranylgeranyl transferase inhibitors I/II (GGTIs I/II) inhibit protein farnesylation and geranylgeranylation, respectively.

The mevalonate (MVA) cascade is a core metabolic pathway that plays a central role in multiple cellular processes by synthesizing sterols and isoprenoids that are essential for cell-signaling, cell membrane integrity, protein synthesis, and cellular respiration [36] (Figure 2).

Isoprenoids biosynthesis is a sequence of cellular reactions leading to the production of two key isoprenoids, farnesyl pyrophosphate (FPP) and geranylgeranyl pyrophosphate (GGPP). Post-translational modification of various proteins, including small GTPases, by FPP and GGPP stimulated interest in their potential involvement with cancer, cardiovascular and neurodegenerative diseases [37,38].

In the first committed step of the mevalonate pathway, acetyl-Coenzyme A (CoA) acetyltransferase (AACT), also called thiolase II, catalyzes the biological Claisen condensation of two acetyl-CoA molecules (derived from the TCA cycle) to give acetoacetyl-CoA. In a second condensation, catalyzed by HMG Co-A synthase, another acetyl CoA group is added to acetoacetyl-CoA to form 3-hydroxy-3-methylglutaryl-CoA (HMG-CoA), which is further reduced into MVA by 3-hydroxy-3-methylglutaryl-CoA reductase (HMGR) [39].

Mevalonate kinase (MK) is the most critical enzyme for the isoprenoid/cholesterol biosynthesis pathway after HMGR, catalyzing the ATP-Mg^2+^ mediated phosphate transfer of mevalonate to produce mevalonate 5-phosphate [40].

MK has been defined as a bottleneck of the MVA pathway, and it is negatively controlled by a feedback loop from geranyl (GPP) and farnesylpyrophosphate (FPP), two crucial downstream intermediates in the final steps of the mevalonate pathway [41].

Next, phosphomevalonate kinase catalyzes another phosphorylation reaction to convert mevalonate 5-phosphate and ATP to mevalonate 5-diphosphate and ADP [42].

In the next step of isoprenoid/sterol biosynthesis, diphosphomevalonate decarboxylase (MDDs) catalyzes the ATP-dependent Mg^2+^ decarboxylation of mevalonate 5-diphosphate to isopentenyl diphosphate (IPP), which is the universal precursor of isoprenoids. IPP can be isomerized to form dimethylallyl pyrophosphate (DMAPP) by IPP isomerase [43,44].

Farnesyl pyrophosphate synthase (FPPS, also known as Farnesyl diphosphate synthase FDPS), a key branchpoint of the mevalonate pathway, catalyzes two sequential condensation reactions of isopentenyl pyrophosphate (IPP), the first with DMAPP, resulting in the production of geranyl pyrophosphate (GPP) and the second with geranyl pyrophosphate (GPP) to produce the C15 farnesyl pyrophosphate (FPP). This product can be processed by geranylgeranyl pyrophosphate synthase (GGPPS), which produces the C20 isoprenoid geranylgeranyl pyrophosphate (GGPP) or exposed to a reductive dimerization reaction by squalene synthase (SQS), which catalyzes the condensation of two identical farnesyl pyrophosphate (FPP) molecules to form C30 squalene [28].

Isoprenoids are required for the production of a variety of compounds, such as ergosterols, dolichols and ubiquinone, as well as for the prenylation of proteins, a key post-translational reaction that is essential for the bioactivity of the proteins. During the post-transcriptional lipid modification of proteins, farnesyl protein transferase (FPTase), geranylgeranyl protein transferase I (GGPTase I) or geranylgeranyl protein transferase II (GGPTase II) enzymes catalyze the addition of a farnesyl or geranylgeranyl lipid group onto a cysteine residue in a characteristic carboxy-terminal motif (e.g., CAAX), producing farnesylated and geranylgeranylated proteins [45,46,47].

Several small GTPases, including members of the Ras and Rho subfamilies, require prenylation to regulate a variety of cell processes that play a crucial role especially in membrane ruffling, membrane targeting and signal transduction [48,49].

In the final steps of cholesterol biosynthesis, squalene monoxygenase (SM) catalyzes the first oxygenation of squalene to (S)-squalene-2,3-epoxide, which is in turn cyclized to lanosterol by an oxidosqualene cyclase (OSC) enzyme. Lanosterol is ultimately converted to cholesterol in numerous oxidations reactions [50].

HMG-CoA reductase and FDPS are two key nodes in MVA pathway regulation with a role in carcinogenesis [36].

In normal cells, cholesterol biosynthesis decreases in the presence of high blood cholesterol levels when dietary intake of cholesterol is abundant and *viceversa* [51].

When the intracellular cholesterol concentration is very high, both HMG-CoA reductase and the LDL receptor are subject to feedback inhibition through the inactivation of the sterol regulatory element-binding protein 2 (SREBP 2) pathway [52].

Statins are HMG-CoA reductase inhibitors that are widely used in the treatment of patients with hypercholesterolemia by blocking cholesterol biosynthesis [53].

Although the benefits of statins are primarily attributed to their lipid-lowering effects, accumulating evidence suggests their efficacy in a variety of tumor cells, including acute myelogenous leukemia [9,54,55].

FDPS is a second key regulatory point of the targeted MVA pathway; small molecules targeting the FDPS signaling pathway have emerged as a promising therapeutic approach for numerous cancers [53,56].

FDPS behaves in a similar way to an allosteric enzyme being negatively modulated by FPP accumulation. This allosteric mechanism is involved in the dynamic regulation of biological prenyl pyrophosphate levels in vivo, as well as in the control of the mevalonate pathway; based on the large implication for cellular activities, human FDPS shows high pharmacological relevance [57].

For example, the nitrogen-containing bisphosphonates (N-BPs) commonly used as anti-bone resorption drugs are potent inhibitors of FDPS. In recent years, there has been growing interest in studying the anticancer effects of bisphosphonates, especially in leukemia treatment [58,59].

The function of oncogenic GTPases depends upon their post-translational prenylation or farnesylation; therefore, bisphosphonates can be used as an indirect strategy to inhibit FDPS activity in tumor cells. Inhibition of FDPS also promotes the accumulation of IPP, which in turn exhibits anticancer effects by activating γδ7 T lymphocytes [60]. Current N-BP drugs are attractive and promising FDPS inhibitors in cancer treatment.

## 3. Regulation of Small GTPase Prenylation in AML

Acute myeloid leukemia (AML) is a hematopoietic malignancy characterized by the clonal expansion and accumulation of immature blood-forming cells in the BM, peripheral blood, and other tissues [61].

Clonal myeloid progenitor cells lose their ability to differentiate into mature blood cells, leading to multilineage cytopenias [62].

One of the molecular hallmarks of AML is the high degree of heterogeneity, since its onset involves a variety of structural chromosomal rearrangements, gene mutations and changes in the expression of multiple genes and microRNAs [63,64].

Many studies have reported that the constitutive activation of FDFT1 in the MAV pathway is also a key factor involved in metabolic dysregulations in acute myeloid leukemia. The high expression of FDFT1 induces the continuous prenylation of Ras proteins, driving tumor progression and invasion [65].

Ras proteins are small guanosine triphosphatases (GTPase) that regulate cell proliferation and differentiation, membrane organization, nucleocytoplasmic transport and cell death. RAS signal pathway disorders are frequently found in myeloid leukemias. The Ras superfamily consists of 167 members, which have been classified into 5 subfamilies (Ras, Rho, Rab, Arf, and Ran), with Ras serving as the founding member [66].

Prenylation is one of the most frequent small GTPase post-translational modifications. In this reaction, a prenyltransferase catalyzes the covalent attachment of a farnesyl or geranylgeranyl lipid to the cysteine within the C-terminal CAAX motif (CAAX box: C is a cysteine residue, A an aliphatic residue, and X is any residue).

Following prenylation, the prenyl-CAAX motif is cleaved by a CAAX prenyl protease 1 (also known as Ste24p) that removes the AAX, resulting in a prenylcysteine as the new C terminus [67,68].

The free carboxyl group of the prenyl cysteine moiety is then recognized by a prenylcysteine carboxyl methyltransferase (pcCMT) that catalyzes the methylesterification of the α carboxyl group [69].

When Ras is prenylated, it associates with the cell membrane, activating signal transduction and cycling between an inactive GDP-bound (Ras-GDP) state and an active GTP-bound conformation (Ras-GTP).

The hydrolysis of GTP to GDP is promoted by GTPase-activated proteins (GAPs), while the conversion from GDP to GTP is catalyzed by guanine nucleotide exchange factors (GEFs) [70].

Guanine nucleotide dissociation inhibitors (GDIs) exert regulatory functions, preventing GEF-mediated nucleotide exchange and preserving the GTPase in an inactive state. Conversely, in the GTP-bound state, GDIs inhibit GTPase activity, blocking GDP dissociation and maintaining the small-GTPase at the membrane.

Additionally, GDI can also mask isoprenoid lipid modification at the C-terminus of the small GTPase, thus, hindering the association of the small GTPase with the membranes.

**Figure 3:**
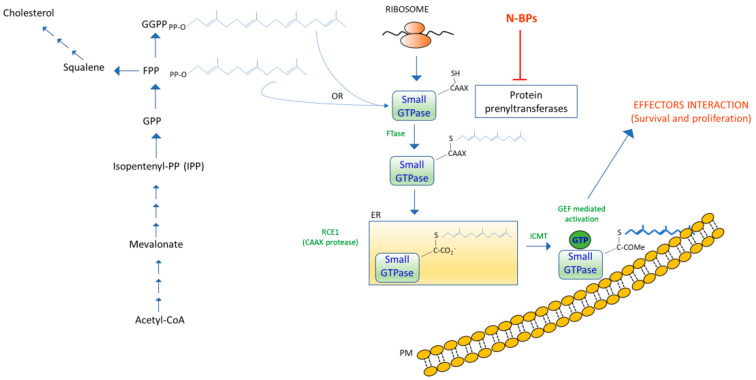
Schematic pathway for the small GTPase post-translational modification of prenylated proteins. Prenyltransferases attach farnesyl or geranylgeranyl lipids, derived from isoprenoid/sterol biosynthesis, to the cysteine within the C-terminal CAAX motif of small GTPase proteins, such as Ras. Nitrogen-containing bisphosphonates (N-BPs) are used to inhibit the prenyltransferases activity. The prenylated proteins undergo further modification on the cytosolic surface of the ER, by intrinsic ER membrane proteins. In particular, Ras CAAX prenyl protease 1 (RCE1) cleaves the AAX, and the resulting free carboxyl group of the prenyl cysteine moiety is methylesterificated by isoprenylcysteine carboxyl methyltransferase (ICMT), in order to target prenylated proteins to cell membrane. ER: Endoplasmic Reticulum; PM: Phospholipid Membrane.

The interaction between Ras-GTP and its specific downstream effectors, such as Rafs and PI3K, stimulate signaling cascades that regulate proliferation, differentiation, and malignant transformation [71,72,73,74] (Figure 3).

Isoprenylation of altered Ras proteins is critical in a large fraction of human tumors, including myeloid leukemias, contributing to the reduction in apoptosis, increase in cellular proliferation and/or survival and adverse outcomes [75,76].

Reducing GGPP and FPP levels by specific inhibitors might affect tumor cell survival as emerged from MAV molecular pathway dissection.

For example, 5-Aza-CdR induced the downregulation of farnesyl diphosphate synthase (FDPS) and farnesyl diphosphate farnesyltransferase, blocking cholesterol biosynthesis.

When acute and chronic myeloid leukemia cells (K562 and HL-60) were exposed to 5-Aza-CdR, this reduced their cellular cholesterol content and showed growth inhibition. This effect was rescued by externally added cholesterol [77].

Additionally, the biological and clinical activity of FTIs, such as tipifarnib, lonafarnib and BMS-214662, have been investigated in hematologic malignancies [78].

In some clinical settings, FTIs have displayed a cytotoxic effect on primary sorted CD34^+^ AML cells, while no survival advantage for untreated AML ≥70 years old was shown [55].

Similarly, GGTI-298 can inhibit cell survival and induce apoptotic cell death in human leukemic cells [79].

The growth in knowledge of the dysregulation in cholesterol metabolism has led to remarkable interest in the anti-cancer properties of bisphosphonates, especially in AMLs.

Bisphosphonates act by targeting FDPS and are usually used to treat bone disease [80].

They could represent an attractive direction for emerging therapies in AML; however, other molecular and clinical investigations are required.

Subsequently, these proteins can be converted from an inactive GDP-bound (small GTPase-GDP) state to an active GTP-bound conformation (small GTPase-GTP) by guanine nucleotide exchange factors (GEFs), which catalyze the conversion from GDP to GTP, to promote interactions between GTP-bound protein and effector molecules, inducing cellular responses of survival and proliferation.

## 4. Bisphosphonates and AML: A Journey through Time

In the last half century, the study of the molecular biology of AMLs allowed the development of chemotherapy and molecular targeted therapy, as well as hematopoietic stem cell transplantation procedures. Despite the availability of many effective treatments, certain patients will relapse following remission. Different factors are associated with the risk of relapse; the minimal residual disease and multiple drug resistance are two key factors. In this context, the molecular target therapy by bisphosphonates is becoming an area of great interest [81].

Bisphosphonates (BP) are a class of calcium-binding drugs commonly used to treat and prevent bone disorders that are characterized by excessive osteoclastic bone resorption, such as osteoporosis and metastatic bone disease [82].

**Figure 4:**
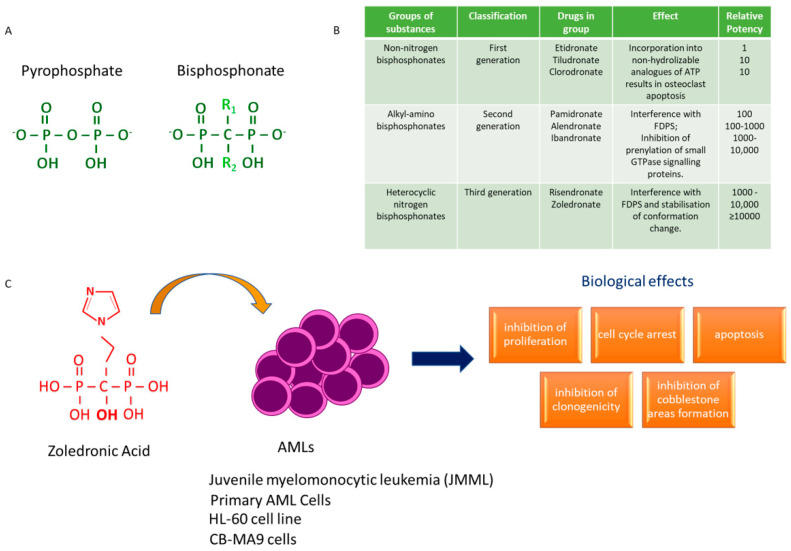
Biological effects of bisphosphonates on acute myeloid leukemia (AML). (**A**) Structural similarities between endogenous inorganic pyrophosphate and bisphosphonate. (**B**) The inhibitory effects of early bisphosphonates, second- and third-generation bisphosphonates on osteoclast differentiation/function via blocking the activity of farnesyl pyrophosphate synthase (FDPS). (**C**) The anti-tumor effects of zoledronic acid on acute leukemic cells.

Bisphosphonates are chemical compound analogues of endogenous inorganic pyrophosphate (PP_i_), in which two phosphonate groups are linked by stable phosphoether bonds to a central (geminal) carbon atom instead of an oxygen atom [83] (Figure 4A).

In the P-C-P backbone structure, two side chains, R1 and R2, are attached to the geminal carbon. The first R group is usually a hydroxyl (-OH) or primary amino (NH2) group, acting as powerful tridentate ligands for calcium (bone hook), while R2 is more varied and is involved in determining the antiresorptive potency of the bisphosphonates, as well as the differences in affinity for hydroxyapatite [84].

Bisphosphonates are usually divided into the following two main categories: nitrogenous bisphosphonates and non-nitrogenous bisphosphonates. Today, after more than three decades of research, BPs can be more specifically classified into three generations according to the chemical structure of the R2 side chain and their molecular mechanism of action [85].

First-generation BPs comprise non-nitrogen-containing compounds carrying minimally modified side chains (e.g., medronate, clodronate, and etidronate) or a chlorophenyl group (e.g., tiludronate). As these molecules are able to most closely mimic pyrophosphate, they can be incorporated into non-hydrolysable analogues of ATP by the class II aminoacyl-transfer RNA synthetases, inhibiting ATP-dependent intracellular events and resulting in osteoclast apoptosis after osteoclast-mediated uptake from the bone mineral surface [86,87].

The second-generation family of bisphosphonates is characterized by the introduction of an N-atom in the side chain (e.g., pamidronate, alendronate, and ibandronate). For the presence of a nitrogen or amino group, these chemical compounds are 10 to 100 times more potent at inhibiting bone resorption in vivo than the simple bisphosphonates [88].

Third-generation BPs are agents that contain a nitrogen atom within a heterocyclic ring in the side chain (e.g., risedronate and zoledronate). These are several orders of magnitude more potent than the first-generation drugs [89].

Unlike early bisphosphonates, second- and third-generation bisphosphonates (alendronate, risedronate, ibandronate, pamidronate, and zoledronic acid) inhibit osteoclast differentiation/function via suppressing the activity of farnesyl pyrophosphate synthase, a critical enzyme for cholesterol biosynthesis in the mevalonate pathway (Figure 4B). As such, bisphosphonates prevent post-translational prenylation of small GTPases, such as Rab, Rac, Rho, which are normally required for many osteoclast cellular activities (including the assembly of stress fibers, plasma membrane ruffling and survival), leading to osteoclast apoptosis [90].

Consistent with the farnesyl pyrophosphate requirement in lipid production, farnesyl pyrophosphate synthase is ubiquitously expressed in mammalian cells; however, nitrogen-containing bisphosphonates are able to induce apoptosis only in osteoclasts [31]. This is due to the ability of bisphosphonates to selectively adhere and remain within bone before endocytosis and within osteoclasts during osteoclast-mediated bone mineral solubilization and organic bone matrix digestion by acid proteases.

Zoledronic acid, a third-generation nitrogen-containing bisphosphonate, is the current standard of care for treating bone metastasis from a variety of solid tumors and multiple myeloma [91,92,93]. ZOL is known to inhibit [3] the FDPS enzyme, resulting in a block in prenylation of small GTPase proteins functionally active in leukemic cells [94].

Recent studies indicate that ZOL also has anti-leukemia activity; however, the detailed underlying mechanism has yet to be elucidated.

Kuroda et al. for the first time in 2003 showed that zoledronate synergistically augments the anti-Ph^+^ leukemia activity of imatinib mesylate, both in vitro and in vivo [95].

Later, ZOL was described as a potent agent in inhibiting the proliferation and clonogenicity of imatinib-sensitive and -resistant CML cells, becoming an attractive candidate for overcoming IM resistance in patients with CML [96].

Emerging studies have also evidenced the surprising inhibitory effects for ZOL on acute myeloid leukemia cells growth in vitro and in vivo, especially when intensive chemotherapeutic regimens have been revealed to be futile in inducing durable remissions. For example, zoledronic acid (ZOL) offers a novel approach to therapy in juvenile myelomonocytic leukemia (JMML), a rare and aggressive myelodysplastic and myeloproliferative neoplasm of early childhood with a poor prognosis. The constitutive activation of the Ras signal transduction pathway is crucial in the development of JMML. Zoledronic acid blocks the abnormal expansion and differentiation of monocytes/macrophages derived from JMML cells of juvenile myelomonocytic leukemia cells, preventing RAS prenylation and activation in vitro. Ohtsuka et al. have demonstrated the capacity of ZOL treatment to reduce the spontaneous colony formation from bone marrow (BM) cells of eight patients with JMML and five healthy control subjects without and with GM-CSF activation, respectively, in a dose-dependent manner. Although ZOL impaired spontaneous differentiation along the monocyte/macrophage lineage of JMML BM cells, granulocyte colonies were formed, suggesting that the constitutively activation of RAS signaling by GM-CSF preferentially stimulates the differentiation of JMML cells along the monocyte/macrophage lineage [97].

In 2012, a clinical trial evidenced the prophylactic use of zoledronic acid to treat and prevent early bone loss in patients with acute myeloid leukemia undergoing allogeneic stem cell transplantation. The treatment with 4 mg of zoledronic acid in 17 AML patients before allo-SCT and for six months after transplantation did not show an increase in the incidence of GVHD (70% vs. 65%) or mortality (47% vs. 47%), compared to patients with AML who received allo-SCT during the same time period (but who were not treated with zoledronic acid). Bone mineral density, measured using dual energy X-ray absorptiometry (DXA) scanning, did not change significantly in any patient over a period of three years (2006–2009), whereas urinary N-terminal telopeptide (uNTX), considered an important marker of bone turnover, progressively decreased over time and serum osteocalcin levels stabilized after six months following transplantation. Importantly, no patient developed osteonecrosis of the jaw, an adverse effect of nitrogen-containing bisphosphonates [93,98,99].

Nowadays, although cellular immunotherapy is a promising therapeutic strategy to treat acute myeloid leukemia, often innate lymphocytes, natural killer (NK) cells and Vg9Vd2 T cells are able to mediate potent graft-versus-leukemia (GvL) effects without clinical evidence for inducing graft-versus host disease (GvHD). In this context, bisphosphonates have immunomodulatory effects that may influence cancer cell progression [100].

As a matter of the fact, N-BP treatment enhances the susceptibility of various cancer cells including AML cells to Vg9Vd2 T-cell-mediated cytotoxicity, causing the intracellular accumulation of MVA cascade intermediate metabolites, such as IPP, which are specifically recognized by Vg9Vd2 T cells [101,102].

Particularly, zoledronate was able to sensitize primary AML cells to Vc9Vd2 T cells, as demonstrated in a comprehensive analysis of clinical, genetic, metabolic, and immunophenotypic features of 19 primary acute myeloid leukemias (AML). N-BP pretreatment enhanced, in a dose-dependent manner, the Vg9Vd2 T-cell cytotoxicity in 50% of the AML samples, whereas 50% of the AML samples were consistently hyporesponsive or refractory to gd T-cell cytolysis. In addition, the ZOL-responsive AML samples showed significantly enhanced HMGCR activity induced by phosphorylation, compared with the hyporesponsive or primarily ZOL-refractory AML samples. Finally, a strong correlation between the activity of the MVA pathway and the sensitivity of the primary AML samples with monocytic or myelomonocytic differentiation to NBP treatment was observed, resulting in the increased susceptibility to Vg9Vd2 T-cell-mediated cytotoxicity compared to the AML samples without monocytic or myelomonocytic differentiation [103].

Recently, some studies evidenced that the treatment of acute myeloid leukemia cells in vitro with BNPs affected their survival and clonal expansion. ZOL treatment inhibited the proliferation and colony formation capacity of HL 60 and adriamycin resistant HL 60 (HL 60/A) cells in a dose- and time-dependent manner, by inducing S phase cell cycle arrest and apoptosis. In this system, ZOL induced apoptosis via the mitochondrial apoptotic pathway by the downregulation of B-cell lymphoma 2 (Bcl-2), upregulation of Bcl-2 associated X protein (Bax) and cleaved poly (ADP-ribose) polymerase (PARP). As ZOL is already available for clinical use, it may be useful as a novel therapeutic agent in addition to chemotherapeutic strategies for AML therapy [104].

The anti-leukemic effects of ZOL have also been evaluated in CB-MA9 cells, an acute myeloid leukemia model obtained from hematopoietic cord blood-derived stem cells transformed with the MA9 fusion gene [105,106,107]. These cells were particularly sensitive to ZOL, displaying inhibition in proliferation, clonogenicity and cobblestone-like structure formation in a dose-dependent manner, compared to normal HSCs and stromal MS-5 cells. The treatment with 20 μM of ZOL inhibited Rap1 prenylation in CB-MA9 cells, compromising the functional activity of the Rac-GTPases family often deregulated in leukemic cells [107] (Figure 4C).

Although the pharmacological action of bisphosphonates lies in the inhibition of bone resorption mediated by osteoclasts, the evidence discussed here proves that they can affect cell viability by altering isoprenoid biosynthesis in cancer cells, including in AMLs.

While many of the studies are in their early stages, bisphosphonates are potentially ideal therapeutic agents to be integrated into conventional chemotherapy regimens for the treatment of AMLs.

## 5. Conclusions

In this review, we discuss the current knowledge concerning bisphosphonates in targeting the mevalonate cascade as a new therapeutic approach in AML, which has not yet been described in this context.

There is now extensive evidence that statins, a class of lipid-lowering medications, have anti-inflammatory and anti-neoplastic properties by blocking the HMG-CoA reductase enzyme in the synthesis of mevalonate.

Although numerous studies over the last two decades have also shown the beneficial effects of bisphosphonates in cancer patients, their effectiveness is still not completely clear with regard to hematological neoplasms.

Bisphosphonates represent an interesting class of pharmacological agents that are able to inhibit bone resorption processes in bone fragility disorders. This effect is of particular clinical relevance in patients with cancer-induced bone disease resulting from the primary disease, especially in MM.

In recent years, experiments on cell-based systems of AML have evidenced BPs as negative modulators of signaling events crucial in promoting clonal expansion and maturation block of progenitor cells in myeloid hematological malignancies.

Here, we firstly summarized the biochemical mechanisms underlying N-BPs’ effects in inhibiting the farnesyl pyrophosphate synthase (FDPS), a key branch-point enzyme in the mevalonate pathway.

Thus, BPs induced a reduction in isoprenoids such as farnesyl pyrophosphate and geranylgeranyl pyrophosphate, preventing small GTPase prenylation. This translational modification negatively modulates cancer key signaling events involved in stimulating cell survival and proliferation.

Next, in this review, we focused on studies that assessed the effects of bisphosphonates in AML over the past twenty years. Moreover, zoledronate was the predominant bisphosphonate evaluated in the majority of research studies on AML model systems.

Zoledronate showed anti-tumor effects on myeloid cell lines and primary leukemia stem cells in vitro. This BP exerted its activity by inducing cellular apoptosis and cell cycle arrest through the perturbation of small GTP-binding proteins activity associated to the MAV transduction pathway.

Today, only a few studies on this subject are available; further research would be useful to help clarify the effect sizes and clinical relevance and significance of BP treatment in AMLs.

In addition, nowadays, few studies have reported the synergistic antitumoral effect of ZOL and farnesyltransferase inhibitor or MAPK or PI3K inhibitors in solid tumors. Indeed, it was reported that the combination of ZOL and R115777, a farnesyltransferase inhibitor (FTI, Washington, DC, USA, Zarnestra), induced apoptosis and growth inhibition in prostate adenocarcinoma cells, but also promoted tumor growth inhibition in vivo in prostate cancer xenografts in nude mice, with a significant survival rate [108].

Moreover, Surmeli et al. revealed that the co-treatment ZOL and serine/threonine phosphatase inhibitors was able to potentiate cytotoxicity and apoptosis in human breast cancer cells, inhibiting the PI3K/Akt pathway [109].

Furthermore, recent studies have shown the synergistic effect of ZOL and trametinib (MEK inhibitor) to inhibit growth and colony formation of MDA-MB-231 breast cancer cells [110], but also potentiate the antitumor activity in KRAS mutant tumors both in vitro and in vivo [111].

The combined treatment of ZOL and farnesyltransferase, or MAPK or PI3K inhibitors, still remains an unexplored field in the context of acute myeloid leukemia and further studies are needed to investigate the potential therapeutic efficacy.

Taken together, these findings support the possibility that third-generation BPs, including zoledronate, can represent an attractive option in the development of new therapeutic approaches for AML treatment and could possibly be integrated into current chemotherapy protocols.

## Abbreviations

Acetil-CoA: acetyl coenzyme A; AACT: acetyl coenzyme A (CoA) acetyltransferase or thiolase II; HMG-CoA: 3-hydroxy-3-methylglutaryl CoA; HMGCR: 3-hydroxy-3-methylglutaryl-CoA reductase; MVA: mevalonate; IPP: isopentenyl diphosphate; IDI: isopentenyl diphosphate isomerase; DMAPP: dimethylallyl pyrophosphate; FDPS: farnesyl pyrophosphate synthase; GPP: geranyl pyrophosphate; FPP: farnesyl pyrophosphate; GGPP: geranylgeranyl-PP; GGPTase I: geranylgeranyl protein transferase I; GGPTase II: geranylgeranyl protein transferase II; geranylgeranyleted proteins (Rho, Rac, Rab, Rap1), SQS: squalene synthase; FPTase: farnesyltransferase.
